# Violence against nurses by patients and visitors in the emergency department: A concept analysis

**DOI:** 10.1111/jonm.13721

**Published:** 2022-07-05

**Authors:** Yongchao Hou, Melissa Corbally, Fiona Timmins

**Affiliations:** ^1^ Emergency Department ShanXi Provincial People's Hospital Taiyuan ShanXi China; ^2^ School of Nursing and Midwifery Trinity College Dublin Dublin Ireland; ^3^ School of Nursing, Midwifery & Health Systems University College Dublin Dublin Ireland

**Keywords:** concept analysis, emergency department, nurse, violence

## Abstract

**Aim:**

This analysis investigates the concept of violence against nurses by patients and visitors in the emergency department. It aims to differentiate, clarify, and clearly identify this specific concept, which will facilitate more apt measurement and reporting, ultimately to contribute violence reduction measures.

**Background:**

Due to contextual factors, occupational risk and patient characteristics, violence against nurses by patients and visitors in the emergency department varies from other types of violence against other health care staff.

**Methods:**

This study employed Walker and Avant's concept analysis technique.

**Results:**

The analysis found that violence against nurses by patients and visitors in the emergency department is primarily an occurrence of interpersonal violence based on the working relationship, whereby the patient and/or visitor becomes an assailant, and a nurse becomes a target in the absence of capable guardianship. There is also an intentional use of physical force or power, which results in or has a high chance of causing harm.

**Conclusion:**

A clearer understanding of the antecedents, attributes, and consequences of violence against nurses by patients and visitors arising from this concept analysis provides a framework that will assist in the understanding, measurement, reporting, and prevention of violence and inform future research.

**Implications for Nursing Management:**

Nursing managers are encouraged to adopt strategies that act on the factors related to attributes and antecedents that will serve to reduce the occurrence of intentional violent acts.

## 1 INTRODUCTION

Violence against nurses by patients and visitors in the emergency department (ED) is on the increase globally (Angland et al., 2014; Copeland & Henry, 2017). Krug et al. (2002) define violence as a complex, multifaceted issue with individual, interpersonal, community and societal aspects, all of which pose a serious threat to health care delivery. Concern about violence has been raised by many professional nursing associations, prompting actions such as nurse surveys, position statements or government lobbying. These associations are increasingly becoming active internationally in terms of encouraging approaches to reduce violence, lowering tolerance and increasing support and prevention (ACEM, 2011). In the United States for example, the Emergency Nurses Association (ENA) carried out a Violence Surveillance Study in 2012 and found that more than half of the ED nurses surveyed reported having been exposed to either verbal or physical abuse within the preceding week (2011 ENA Emergency Nursing Resources Development Committee et al., 2012). They also prepared a position paper on this (Emergency Nurses Association, 2006).

More recently, Australian ED nurses took to protesting about rising levels of ED violence (Anonymous, 2017). In the United Kingdom, the Royal College of Nursing (RCN) (2017) also voiced concern at a reported 28% occurrence of physical violence among 6000 nurses surveyed. They welcomed the Assaults on Emergency Workers (Offenses) Bill 2017‐19, a Private Member's Bill, that introduced a new offense of assaulting an emergency worker and new sentencing guidelines. The RCN outlined their support for the Bill and suggested more far‐reaching measures to protect health care staff from violence and aggression (RCN 2017). Similarly, the Royal College of Emergency Medicine (RCEM) (2019) voiced concern about the rising violence against health care workers and especially those working in the ED.

However, the prevention of violence in the ED is complex, and while legislation and greater action towards perpetrators is welcome, a greater understanding is needed of the phenomenon so that prevention tactics can be meaningfully utilized. Most studies in the field of contributing factors of violence against nurses in the ED by patients and visitors have focused on the behaviors and characteristics of assailant, identifying those at risk and targeting these (Carver & Beard, 2021; Kleissl‐Muir et al., 2019; Ramacciati et al., 2017; Terry Ferns & Rew, 2005; Zafar et al., 2013). However, deliberate planned attacks of violence on ED staff are rare, and rather the most common occurrences are due to environmental aspects of ED, and its day‐to‐day management (Spelten et al., 2020; Timmins & Timmins, 2021). Indeed, recent study findings resulted in a new conceptualization of ED violence, which gave an equal weighting to the actions of the assailant and environmental/organizational factors. This approach considers the potential contribution of the ED setting to the occurrence of violence (Ramacciati et al., 2017), something which is interesting, but also controversial. However, other studies support this finding (Morphet et al., 2014; Ogundipe et al., 2013). For example, Boyle and Hassett‐Walker (2008) highlighted the need to understand the assailant in the context of the circumstances and the ED setting to more fully understand the violent episode. Unlike many other health care settings, ED settings are often a public facing, open access entry point for health care services at hospitals, operating over a 24‐h period. Specific environmental issues, which are unique to the EDs, and feature globally, which include 24‐h accessibility, overcrowding, long waiting times, “frustration of patients,” and sometimes inadequate security systems (Al‐Qadi, 2020; Ogundipe et al., 2013), are all linked to ED violence. ED violence is also often associated with patients who are intoxicated, or who present with mental health crises (Timmins & Timmins, 2021). Long waiting times, notoriously associated with EDs, both evoke and exacerbate this situation (Timmins & Timmins, 2021). Therefore, consideration of environmental factors can help to understand the phenomenon of violence in the ED.

Many associations call for zero‐tolerance policies on violence in the health care setting (ACEM, 2011; Hassankhani & Soheili, 2017; NSW Health, 2003). The American Nurses Association (2015) for example stated that “the nursing profession will not tolerate violence of any kind from any source.” This is a useful turning point as historically, nurses were taught to ignore and sacrifice their own feelings of fear or anger, for the “greater good” of patient welfare (Lanza, 1984). Thus, the increasing recognition of the intolerability of violence towards nurses is welcome but also needs comprehensive and holistic initiatives to tackle it. The goal of zero tolerance on its own is not enough, and indeed is overly ambitious given the high‐risk nature of ED nursing work where there is a high probability of violence occurring (Copeland & Henry, 2017), and the inextricable link between ED violence and the presenting conditions (such as drug and alcohol intoxication, head injuries, and other issues that affect cognition). As such, a multifaceted approach to the prevention of ED violence requires acknowledgement of this high occupational risk of ED nurses so that appropriate measures may be taken to reduce the incidence of violence. The scope of practice and code of conduct of ED nurses also needs careful consideration, as they are required to provide respectful care to all patients. A dilemma and challenge may exist therefore in how best to deal with episodes of violence. Patients in need of care cannot be turned away, for example, even if their behavior is out of control (Aljohani et al., 2021). It would also be unethical for ED nurses stand in the way of treatment those requiring medical attention, by for example prioritizing the reporting and possible arrest of a violent patient.

Tackling this issue also requires clear documentation to support investigation and action in specific events but also to understand the scope of the problem. However, there is a lack of documentation of violent events in health care generally, due to underreporting (Aljohani et al., 2021; Christensen & Wilson, 2022; Huang et al., 2022; Stene et al., 2015). Barriers to reporting might arise from existent discrepancies between what health care organizations encourage nurses to report and what nurses actually report, perhaps due to nurses' conflicting obligations and ethical concerns about doing no harm to the patient (Buterakos et al., 2020). The reporting behavior of ED nurses is also informed by how they define and understand violence in this context. ED nurses assign different meanings of violence based on the intention of the assailant and harm it brought to the victim (Ashton et al., 2018). Take an example a patient who has delirium and is attempting to hit an ED nurse. This will likely be interpreted very differently than a visitor who is attempting to do the same thing. The former are classified as problem behaviors by nurses rather than acts of violence (Erickson & Williams‐Evans, 2000; Richardson et al., 2018). This is consistent with the definition used by the World Health Organization (WHO), which associates intentionality with the committing of the act itself, irrespective of the outcome it produces (Krug et al., 2002). Besides, there are also divergent views on the perception of harm. If no nurse is harmed during the patient‐related violence, an event might not meet an individual's threshold for workplace violence, which requires reporting (Christensen & Wilson, 2022; Huang et al., 2022). However, some might argue that the potential for harm exists, regardless of whether actual harm occurred, which should therefore necessitate reporting (Copeland & Henry, 2017). Similarly, the WHO highlights that defining consequences solely in terms of harm or death thus limits the understanding of the full impact of violence on individuals, communities and society at large (Krug et al., 2002). Therefore, clarifying these nuances is necessary for understanding the concept of ED violence.

While there are specific references in the literature aimed at specifically defining violence as a concept in nursing contexts (Ghosh et al., 2019; Murray et al., 2020), there is little specific understanding in relation to ED in particular. This is important, given the particular highly charged ED context within which such behaviors take place. Moreover, violence is also understood more generally as an umbrella term by researchers encompassing verbal abuse, physical assault, or the witnessing of either of these acts (Abou‐ElWafa et al., 2015; Ferns, 2005; Gill et al., 2002; Lancman et al., 2013; Ramacciati et al., 2019), which leads to difficulty understanding the key issues at stake or taking action as a result of the findings. Other literature sources define violence such that it also includes all and every incident of violence, including instances of aggression, bullying, intimidation, harassment, and workplace incivility by staff members (DeWall et al., 2011). As such, a more specific explanation that focuses on patients/visitors only and clearly identifies the consequences of violence in the ED would be helpful. Therefore, an operational definition of violence against nurses in the ED by patients and visitors is needed to address specific issues of understanding given the breadth of definitions and scope within the existing literature (Aljohani et al., 2021), and the specific nature and context of ED violence, and limited attention to this area. The goals of this conceptual analysis were twofold:

1. To explore the defining attributes, antecedents, consequences, and empirical referents of violence against nurses by patients and visitors in ED settings.
2. To formulate an operational definition of the violence against nurses by patients and visitors in ED settings.

## 2 METHODS

### 2.1 Concept analysis approach

Analyzing violence as a concept as it relates to ED nursing practice is important as there are specific nuances within the ED nursing context that affect its manifestation, consequences, and ultimately management. The context of the ED is highly emotionally charged and is much more public facing and exposed than most health care environments; thus, analysis within this context is important. It is the site of most reported health care violence, and as such, a nuanced understanding would be immensely beneficial to our understanding and prevention. Without this, there is risk of using broad sweeping definitions that do not fully apply to this area.

Walker and Avant's (2011) method continues to be by far the most popular method of concept analysis among nursing scholars due to its applicability to nursing contexts (Janice Penrod, 2005; Paley et al., 2007). Although the context bound nature of concepts have been identified as potentially limiting (Paley et al., 2007), we argue that in this instance, violence and the context of ED are inextricably linked and having a context free definition of such a context bound phenomenon would be counterproductive and too abstract for practical application for ED nurses and their managers. Furthermore, there are multiple applications and uses of conceptual analysis in the literature that explore commonly understood concepts within particular contexts. The purpose of this concept analysis was to explore the defining attributes of ED violence to nurses by patients and visitors in the ED. We defined the aim of our analysis, as defined all uses of this concept, and identified its defining attributes in a model case, borderline case, contrary case, summarized its antecedents and consequences, and defined its empirical referents. Thus, the first two stages of the concept analysis process (i.e., one—selecting a concept; two—determining the purposes of analysis) are thus understood. The findings from the remaining stages are presented below.

### 2.2 Data sources

**FIGURE 1:**
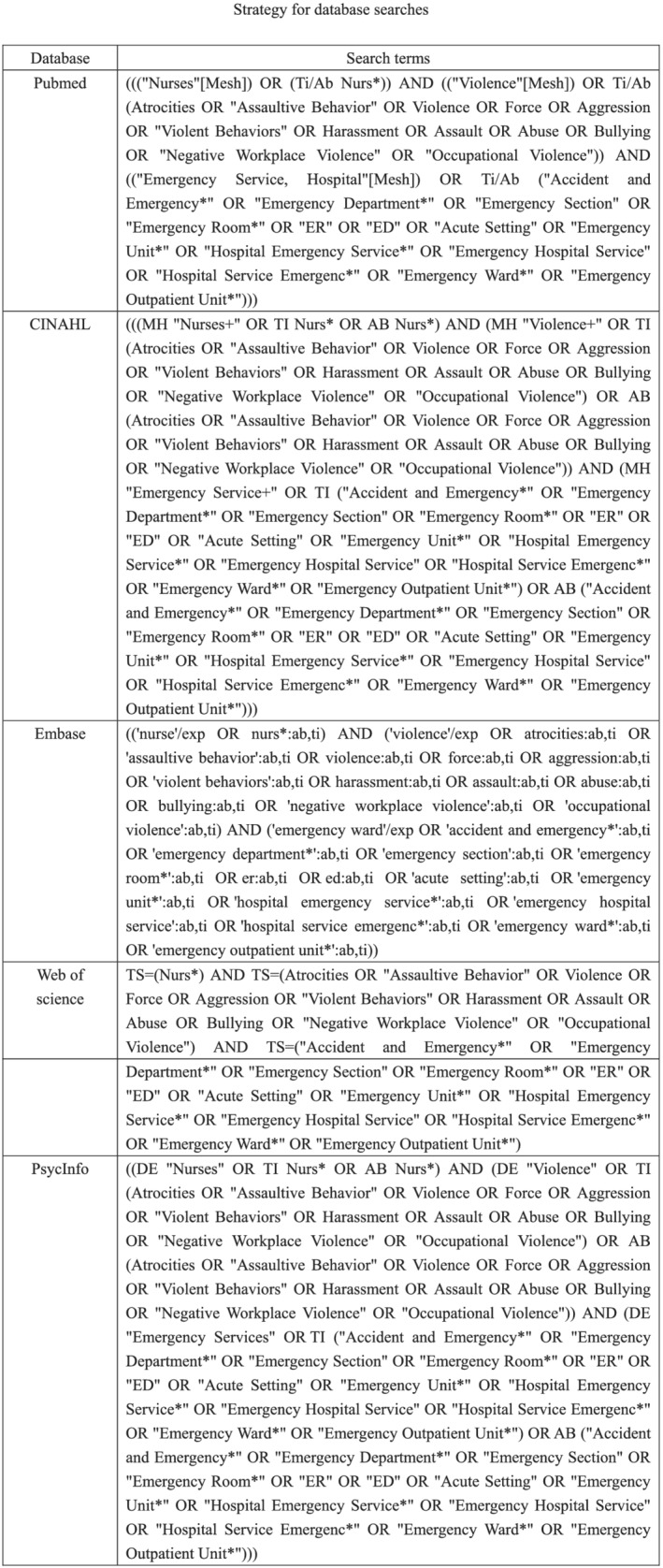
Strategy for database searches

Determining all uses of the concept constitutes the third stage of the concept analysis process. In addition to exploring common definitions using dictionary sources, we searched the empirical literature to better explore the concept of violence against nurses by patients and visitors in ED settings. Pubmed, Embase, Web of science, CINAHL, and PsycINFO databases were systematically searched without any limits on the year of publication. Article titles, abstracts, and full‐text reports were searched with medical subject headings (MeSh) and other search terms (Figure 1). In addition, the references of relevant studies and journals were manually searched to identify any other articles appropriate for inclusion in this comprehensive analysis (Magarey, 2001).

**FIGURE 2:**
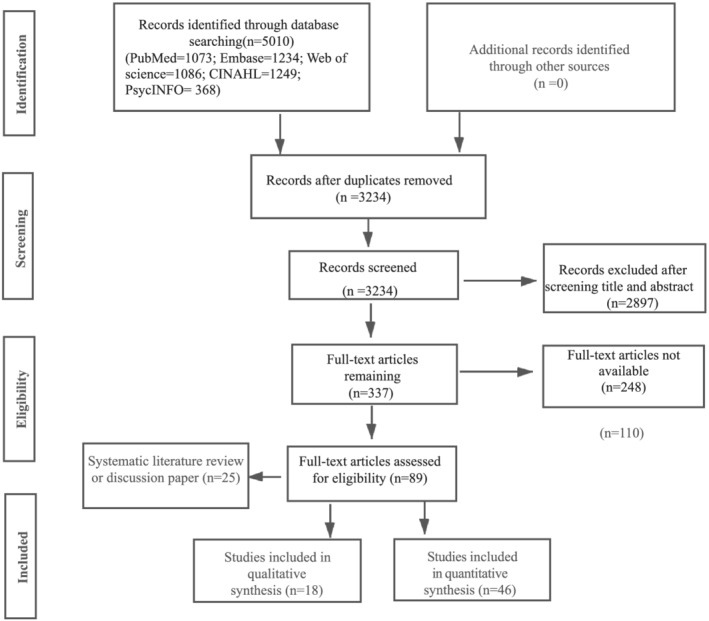
PRISMA flow diagram of the literature search

In addition to the use of dictionary and gray literature sources to support understanding of definitions, empirical articles were eligible for inclusion if they were original research articles, systematic reviews, case studies, or secondary analyses published in peer‐reviewed journals that contained the term “violence” or related terms including “workplace violence” and “patient‐related violence” used in an ED context. Eligible research included qualitative, quantitative, mixed‐methods, and review articles published in English. Articles not meeting these criteria were identified by screening the titles and abstracts of potentially relevant studies, followed by a full‐text review to evaluate contextual details consistent with this concept. A total of 89 articles were reviewed, and informed the concept analysis, and the results are presented below (Figure 2).

## 3 RESULTS

### 3.1 Definitions of violence

The Oxford English Dictionary and Webster's Dictionary define violence as the exercise of physical force with the intent to injure or damage persons or property, categorizing violence as the use of physical force with intent to do harm. This singular understanding is difficult to apply in the ED context, as intentionality is not always present (Gates et al., 2006), and violence is not necessarily only associated with physical force. Indeed, a variety of acts can be constituted as violent (Khan et al., 2021) such as any verbal or physical action directed against ED nurses (Gates et al., 2006). However, although Gates et al. (2006) describes acts as violent whether they are intentional or not, intentionality is an important aspect of how violence is conceptualized within international nursing policies (e.g., the Canadian Nurse Association, 1996; and the WHO, 2002). Generally, within health care, it is the patient's intention that renders whether an act is perceived as violent by staff. Indeed, the WHO (2002) defined violence as “the *intentional* use of physical force or power, threatened or actual, against oneself, another person, or against a group or community, that either results in or has a high likelihood of resulting in injury, death, psychological harm, maldevelopment or deprivation” (emphasis not original). This notion of intent may then serve to discount the violence experienced by ED nurses from patients who have cerebral injury and mental health issues or who are intoxicated, perhaps serving to further encourage under reporting and dismiss the effects. As such, the act of one individual severely harming another may not inherently be violent if the injured party had no intention to cause such injury.

A further issue with these definitions is the outcome (of violence) as injury or high risk of injury because actions can be violent, even when no damage to persons or property occurs, if there has been intent to do harm (Heitmeyer & Hagan, 2003). Therefore, violence can be conceptualized as resulting from the intent to cause harm irrespective of the ultimate outcome. The importance of intentionality is thus one of the most central and complex facets of the definition of violence.

Additionally, while the use of physical force appears to be consistent with many definitions and original understandings, and indeed at an etymological level the term is derived from “vis” (force) and “latus,” which is the past participle of “fero” (to carry) meaning to carry force (towards something) (Springer & Le Billon, 2016), violence is not necessarily only physical. A variety of acts can be constituted as violent, resulting in a variety of physical and psychological outcomes (Khan et al., 2021). This raises the question of the precise link between violence in the occurrence of harm or injury, and makes it unclear as to whether the incidence of harm or the foreseeable risk of such harm is an intrinsic feature of any violent act (Shi et al., 2017). Consequently, violence may be understood as a “spectrum of behaviors ranging from passive aggression to homicide” (Gormley et al., 2016) to capture the breadth of violent acts. Thus, greater clarification of the attributes, antecedents and consequences of ED violence against nurses by patients and visitors will help improve understanding of this complex concept.

### 3.2 Defining attributes

Demonstrating a cluster of attributes is a core feature of concept analysis (Walker & Avant, 2011). Certain attributes can be used to develop a definition of violence against ED nurses by patients and visitors that is more realistically reflective of how policymakers and nurses use the term in the emergency department. Six common attributes including three core critical attributes and three others were identified from this analysis including: The three critical attributes are as follows: (1) A “patient and/or visitor who becomes an assailant” (Abdellah & Salama, 2017; Berlanda et al., 2019; Cannavo et al., 2019; Davey et al., 2020; Pich et al., 2011; Renker et al., 2015; Spelten et al., 2020); (2) the presence of “nurses who became a suitable target” (Ashton et al., 2018; Han et al., 2017; Kennedy & Julie, 2013; Renker et al., 2015); and (3) “the absence of capable guardianship in ED setting” (Hamdan & Abu Hamra, 2015; Renker et al., 2015). These three attributes are the most critical attributes because violent behavior requires a concurrence in space and time of a likely, a suitable target, and an absence of capable guardianship according to the key insight of routine activities theory (Cohen & Felson, 1979). In the concept of patient‐related violence against nurses in the ED, patients and nurses change their roles to become assailants and victims, leading to violent behaviour occurring where guardianship is lacking. Guardianship can be interpreted in many ways including a person or an object that is effective in protecting the target and deterring attack to occur. Three other important critical attributes also emerge, namely, the demonstration of a “a work relationship between an assailant and a suitable target” (Sonis et al., 2018), which is configured as the fourth attribute. According to the typology of violence, patient‐related violence against ED nurses is an interpersonal violence that occurs in the workplace, thus demonstrating a work relationship between an assailant and a suitable target is an important attribute (Krug et al., 2002; Canadian Nurses Association, 1996; Copeland & Henry, 2017). There is also “the intentional use of physical force or power” (Krug et al., 2002; Maguire et al., 2018; Murray et al., 2020; Khan et al., 2021), as the fifth attribute. In addition to the phrase “use of physical force,” the use of the word “power” broadens the nature of a violent act and expands the conventional understanding of violence to include verbal abuse, such as threats and intimidation, as well as sexual and psychological abuse (Al‐Qadi, 2020; Ashton et al., 2018; Krug et al., 2002; Partridge & Affleck, 2017; Pich & Kable, 2014). A sixth and final attribute emerges as either resulting in “or has a high chance of causing harm” (Aljohani et al., 2021; Krug et al., 2002; Sonis et al., 2018; Wolf et al., 2020).

### 3.3 Antecedents

According to the literature, antecedents to violence against nurses in the ED by patients and visitors include factors related to patients and/or visitors, factors related to nurses, and factors related to ED setting. These will now be described.

#### 3.3.1 Factors related to patients and/or visitors

People visiting ED can be influenced to commit violence relatively easily. First, the trigger to incite violent behaviour of patients and/or visitors is being aware of the fact that one is deprived (Tadros & Kiefer, 2017). Patients and/or visitors are often in a state of severe mental distress and frustration (Abdellah & Salama, 2017) owing to the patient's perceived urgent medical problem, pain, fear of the unknown, and long wait (Davey et al., 2020; Landau & Bendalak, 2008). This in turn may lead to impaired rational judgment, increasing the likelihood of violence of patients and and/or visitors (Landau & Bendalak, 2008). Some patients may tolerate and cope with their fears, or even with their anger, and be cooperative in the hopes that they can resolve their problems, whereas others would treat the emergency department experience as a trigger and they would be more apt to become violent or abusive as a means of coping with their frustration and perceived deprivation (Lau et al., 2012). Therefore, when ED patients and/or visitors feel deprived, they may revolt and become violent. They may view it as “good” violence, or as a means to an end, that end being resolution to the problem that brought them to the ED, or perhaps a perceived punishment for the nurse who they perceive has not cared for them appropriately. Second, subcultures' adaptations of the “machismo” image is another trigger to incite violent behaviour of patients and/or visitors (Cannavo et al., 2019; Cikriklar et al., 2016). Visitors were more likely to be involved in episodes of violence in non‐Western studies (Ashton et al., 2018; Hamdan & Abu Hamra, 2015; Krug et al., 2002; Nithimathachoke & Wichiennopparat, 2021; Tadros & Kiefer, 2017). Typically, this involved male assailants and the actions were against female nursing staff. This was thought to be related to the roles played by males in these cultures and the fact that violence towards women is more prevalent in countries such as Turkey and Iran due to male dominance in these cultures (Ayranci, 2005; Krug et al., 2002; Pich & Kable, 2014). Third, exposure to alcohol, drugs, and violent acts observed on popular media (and perhaps normalized as acceptable behavior in the circumstances, despite these being fictitious accounts of behavior) may bring a high risk for violent behaviour (Cikriklar et al., 2016; Harthi et al., 2020).

#### 3.3.2 Factors related to nurses

##### The vulnerability of the ED nurses

In the Routine Activity's theory, Cohen and Felson (1979) demonstrated that the “inertia” factor plays an important role in which people become a suitable target in the violence event, which refers to how difficult it is to move or transport an object based on its natural attributes: weight, height, strength (Landau & Bendalak, 2008). In other words, it is much easier to attack the female nurse, whereas it is quite difficult to take action against male staff. Despite the fact that in terms of age, educational background, and professional experience, the profile of ED nurses in studies that explore violence varies, several studies have found that female nurses were more vulnerable to attack in the ED (ALBashtawy et al., 2015; Cannavo et al., 2019; Hyland et al., 2016; Johnsen et al., 2020; Landau & Bendalak, 2008; Partridge & Affleck, 2017; Ramacciati et al., 2015; Zhang et al., 2017).

##### The visibility and accessibility of the ED nurses

How suitable a target of attack is also depends on the visibility and accessibility of the ED nurses in the workplace (Cohen & Felson, 1979). Obviously, ED nurses in their position act as the major “gatekeepers” to the ED, in a public space that everyone can enter and are therefore easier to target compared to other departments in the hospital or health care setting (Ferri et al., 2020). Besides, working more weekly hours in the public domain also increases nurses' exposure to potential assailants and the higher the nurse's weekly workload in this context, the greater her/his chances of victimization to violence (McGuire et al., 2021; Partridge & Affleck, 2017).

#### 3.3.3 Factors related to ED settings

Owing to lack of guardianship, which can be a person or an object that is effective in deterring violence to occur (Boyle & Hassett‐Walker, 2008), the ED has been recognized as an environment with a high potential for stressful interactions that are aggravated by a vicious cycle of misconceptions, frustration, and anger (Li et al., 2019). Factors specific to the absence of capable guardianship in ED perceived to have contributed to episodes of violence included easy access to the department, over crowdedness (Ashton et al., 2018; Hamdan & Abu Hamra, 2015; Sonis et al., 2018), long waiting times, slow response times from security, and a lack of metal detectors (Gillespie et al., 2017; Lau et al., 2012; Renker et al., 2015).

### 3.4 Consequences

The consequences of violence are far‐reaching in terms of nurses and patient outcomes. These may take the form of physical, emotional/psychological, and professional consequences. Physical consequences range from minor injuries to various body parts to severe disablement and death (Hassankhani et al., 2018; Zhang et al., 2017). Emotional and psychological consequences of violence are complex including posttraumatic stress disorder (PTSD), depressive symptoms, anxiety, feeling unwell and lacking self‐esteem, and undesirable emotions. Professional consequences of violence manifested as dissonance between nurses' professional role as caregiver and the role as crime victim, reduced job satisfaction (Kowalenko et al., 2013), loss of self‐confidence, avoidance of patients, decreased productivity, isolation from team bonding, burnout (Jimenez et al., 2019), sick leave absence, transfer to another position, duty change, and leaving the profession (Ferri et al., 2020; Han et al., 2017).

### 3.5 A model case

Model cases of a concept are instances that clearly embody all the critical aspects of that concept (Walker & Avant, 2011). Below, we present a clear‐cut model case of violence against a nurse in the ED.

At 1.30 am, the triage area of the ED was still very crowded. Three nurses were on duty in the triage area. They occupied the triage area along with a crowd of patients and their accompanying families and visitors. The triage desk directly faced the main entrance to the ED. It was specifically designed in this way so that people who come to the ED go straight to the triage desk. A nurse in the ED was caring for a patient suffering from abdominal pain, nausea, diarrhoea, and pyrexia in the examination room located behind the triage desks. She attempted and failed to insert an intravenous cannula to commence an intravenous infusion. The patient became angry and began to shout at the nurse, and then pushed a large metal mobile stand (used to hold intravenous fluid) into her. The nurse sustained an injury and ran out of the room crying.

This model case exhibits all the key attributes of violence against a nurse by a patient in the ED. The violence occurred in the ED without capable guardianship, the patient became an assailant and attacked the nurse who was the suitable target as she was delivering the care for them. The assailant and the victim had a nurse–patient relationship, and the use of deliberate physical force together with verbal assault resulted in an injury coupled with psychological upset.

### 3.6 A contrary case

Walker and Avant (2011) stated that a contrary case helps to improve the clarity of the concept under scrutiny by presenting a case that does not reflect the concept.

A nurse was caring for a patient suffering from abdominal pain, nausea, diarrhoea, and fever in an ED setting. The nurse took the patient's blood pressure and temperature and then explained to the patient that they will need to undertake a blood glucose measurement test. The nurse says, “I am afraid I have to prick your finger and take a drop of blood to test your blood sugar level.” “Okay,” the patient agrees, and the nurse undertakes their work without any issue arising.

In this case, no physical or verbal assault occurred, there was no actual or potential harm, and there was no victim or assailant such that no violence occurred in this scenario.

### 3.7 A borderline case

Borderline cases are examples, which comprise almost all the aspects of a given concept, but with one attribute that differs substantially from that conceptual framework (Walker & Avant, 2011). An example is given below:

A nurse was caring for a motorcycle crash victim with a traumatic brain injury of the frontal lobe in the ED. The patient is agitated and exhibited reduced awareness of their environment. Sometime later, when washing the patient, the nurse is suddenly hit in the face with the patient's fist. The nurse experiences facial swelling and redness.

This borderline case encompasses almost all of the attributes of violence against nurses by patients and visitors in the ED, including a patient–nurse relationship, the ED setting, the use of physical force towards a nurse by a patient, and a resultant injury. However, as the patient had no intention to cause injury, this does not meet the criteria to be defined as an act of violence.

### 3.8 Empirical referents

**FIGURE 3:**
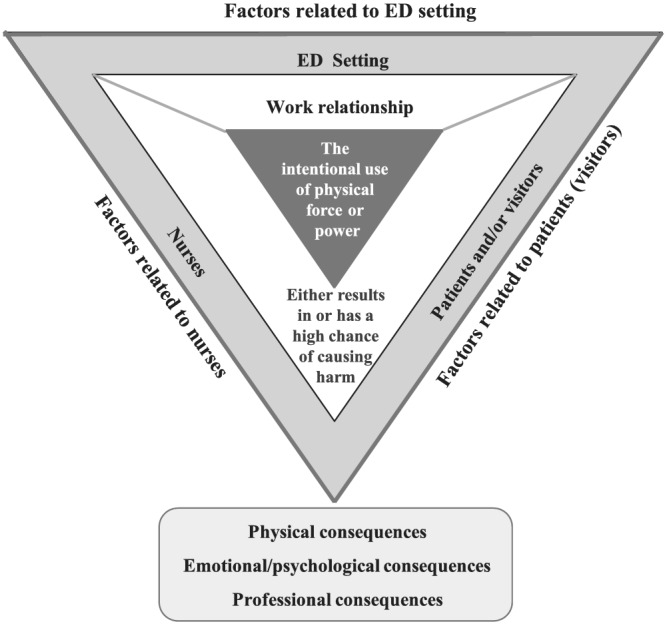
The attributes, antecedents, and consequences of violence against nurses by patients and visitors in the emergency department

The last step when conducting a conceptual analysis is the determination of empirical referents for critical attributes of a given concept. These referents are classes or categories of phenomena that provide evidence of the existence of that concept (Walker & Avant, 2011). For the purposes of this concept analysis, ED violence is intentional behaviour, resulting from ED environmental factors, that involves physical force and/or verbal force to the nurse, by patients or visitors and results in or has a high chance of causing harm. An outline of this concept map is provided in Figure 3.

## 4 DISCUSSION

A safe practice environment for nurses is critical to the integrity of the health‐care system and to the quality of patient care. However, the ED has been identified as a workplace with one of the highest risks of violence towards staff (Abou‐ElWafa et al., 2015; WHO, 2012). Within this context, ED nurses are at a higher risk of verbal or physical violence compared with other health care professionals, or those working in other departments (Ferri et al., 2016; Kowalenko et al., 2012; Morphet et al., 2014; Wu et al., 2019). This concept analysis of violence against nurses by patients and visitors in ED reveals that violence is intentional behaviour, resulting from ED environmental factors, that involves physical force and/or verbal force to the nurse, by patients or visitors and results in or has a high chance of causing harm. This conceptualization supports, for the first time, a definition of ED violence that includes the effect of the environmental and organizational factors that have been identified by previous researchers (Ramacciati, Ceccagnoli, Addey, Lumini, & Rasero, 2018; Ramacciati, Ceccagnoli, Addey, & Rasero, 2018) but not categorized within current understandings or definitions of ED violence. This will contribute to a more holistic approach and understanding of ED violence for both research and practice, that considers the potential contribution of the ED environment to the occurrence of violence (Ramacciati, Ceccagnoli, Addey, Lumini, et al., 2018; Ramacciati, Ceccagnoli, Addey, & Rasero, 2018), thus assisting with measurement and prevention.

The fact that the ED is an environment with high likelihood for violence, harm prevention is an important factor for nurse managers to consider. Having a clear understanding of how nurses conceptualize such violence is integral to its management and prevention. This concept analysis suggests that although the term violence may be appropriate in a technical sense, it may not be effective or clinically useful at a practical level given that neither nurses nor patients typically utilize the term violence when referring to these behaviors, given that they are often understood as implicit effects of a high risk job (Maguire et al., 2018; Ramacciati, Ceccagnoli, Addey, Lumini, et al., 2018; Ramacciati, Ceccagnoli, Addey, & Rasero, 2018) and this may be a consequence of specific social norms (Buterakos et al., 2020).

Developing new awareness and understandings of ED violence towards nurses involves identification, recognition, and reporting of situations where there is intentional verbal and physical force that is potentially harmful while at the same time differentiating this from situations where there is no intent. This may be complex for ED nurses and will require effective support from nurse managers and policy, to be able to unpack, understand, manage, and report when an acts of violence occurs that are not intentional. Certainly, there may still be consequences for the ED nurse, including possible physical and psychological sequelae, but the intervention from a reporting and support perspective may be different. Additionally, much of the violence experienced by ED nurses arises from situations of patient mental health crises and/or intoxication, and it will be morally and ethically challenging for nurses in practice to elucidate whether there was intent. Thus, the complex nature of violence against nurses by patients and visitors in ED settings and its resultant consequences underscore the importance of nurses being able to understand and report instances of violence (Ramacciati et al., 2017) and also the need to develop more sensitive measurement tools that will clearly identify environmental triggers.

However, there is also evidence suggesting that nurses lack information, experience, and interest in the early warning signs of violence and associated prevention strategies (Koller, 2016; Presley & Robinson, 2002). Indeed, nurses may not even recognize violent acts as violent, choosing to accept violence from patients and visitors as part of the job. ED nurses may also find this concept analysis to be of value therefore as a means of better recognizing potential triggers for violence in ED settings and use these to implement individualized comprehensive strategies to manage and respond to such violence (Copeland & Henry, 2017).

Instances of violence in health care settings are also thought to be an important factor associated with challenges in the recruitment and retention of nurses, increasing the risk of nursing shortages and negative outcomes for both health care organizations and patients. Environments that encourage violence prevention and management and that are actively supported by local leadership for this are essential to minimize and mitigate instances of such violence (Wax et al., 2016). By better understanding the violence against nurses by patients and visitors in EDs, policymakers and nurse managers may be better positioned to recognize the need for education and training related to such violence (Stene et al., 2015), and may be more likely to enact policies that are necessary to maintain a safe and healthy working environment (Gerdtz et al., 2013). By establishing a culture of justice and ensuring the rights of nurses to privacy when reporting incidents, injury care, debriefing, and counseling, nursing leadership have the potential to largely overcome many of the major barriers to violence reform in ED settings (Chappell, 2015; Sharifi et al., 2020).

For researchers, accurately comparing the incidence of ED violence across countries remains challenging owing to reporting deficiencies, research design and methodological issues, and inconsistencies with respect to how violence is defined (Maguire et al., 2018; Ramacciati, Ceccagnoli, Addey, Lumini, et al., 2018; Ramacciati, Ceccagnoli, Addey, & Rasero, 2018). More rigorous data‐driven analyses and improvements in incident reporting, risk surveillance, successful mitigation, and the evaluation of violence‐related interventions will only be possible if standardized definitions are used (Wolf et al., 2020). As such, this concept analysis has key implications for research in this area for the future. Consistently conceptualizing the violence against nurses by patients and visitors in ED settings represents a preliminary step in the context of theory development with the goal of refining and testing a conceptual model (Ghosh et al., 2019). Further research will be critical to establish the most effective prevention and mitigation strategies, to define educational priorities, which may aid nurses in recognizing high‐risk patients and scenarios, and to determine the appropriate conditions for proactively reducing these instances of workplace violence (Azami et al., 2018).

## 5 IMPLICATIONS FOR NURSING MANAGEMENT

The ED environment in which nurses work with patients and visitors from all aspects of society provides high‐level care in challenging situations. Sadly, nurses practicing in this context run a high risk of being intentionally harmed by patients and visitors compared with other health care settings, and the potential for actual and cumulative harm experienced by staff is high. It is vital that the violence against nurses by patients and visitors in ED settings be understood, and the model cases discussed in this article are used as valuable tools for distilling the key aspects of such violent episodes. By considering the attributes, antecedents, and consequences of the violence against nursing staff by patients and visitors in ED environments, it may be possible to better aid in protecting against such violence through administrative, educational, environmental, and security interventions. However, such violence remains a highly complex and elusive complex, and there is thus an urgent need for the design of additional measurement and prevention strategies, including the education of nursing staff regarding approaches to recognizing potentially high‐risk patients and the appropriate conditions for proactively intervening to reduce violent occurrences. This concept analysis provides a framework that can be used to clarify patient‐related violence towards ED nurses. Nurse managers should adopt strategies that act on the factors related to attributes and antecedents and thus increase understanding of the factors that contribute to the occurrence of ED violence, while at the same time seeking to directly address these attributes in order to reduce the occurrence of intentional violent acts.

## CONFLICT OF INTEREST

No conflict of interest has been declared by the authors.

## Data Availability

All data are available upon reasonable request.

## References

[jonm13721-bib-0001] 2011 ENA Emergency Nursing Resources Development Committee , Crowley, M. , Brim, C. , Proehl, J. , Barnason, S. , Leviner, S. , Lindauer, C. , Naccarato, M. , Storer, A. , Williams, J. , & Papa, A. (2012). Emergency nursing resource: Difficult intravenous access. Journal of Emergency Nursing, 38(4), 335–343. 10.1016/j.jen.2012.05.010 22770395

[jonm13721-bib-0002] Abdellah, R. F. , & Salama, K. M. (2017). Prevalence and risk factors of workplace violence against health care workers in the emergency department in Ismailia, Egypt. The Pan African Medical Journal, 26, 21. 10.11604/pamj.2017.26.21.10837 28451000 PMC5398248

[jonm13721-bib-0003] Abou‐ElWafa, H. S. , El‐Gilany, A. H. , Abd‐El‐Raouf, S. E. , Abd‐Elmouty, S. M. , & El‐Sayed Rel, S. (2015). Workplace violence against emergency versus non‐emergency nurses in Mansoura university hospitals, Egypt. Journal of Interpersonal Violence, 30(5), 857–872. 10.1177/0886260514536278 24970863

[jonm13721-bib-0004] ACEM . (2011). Policy on violence in Emergency Departments. Retrieved from https://acem.org.au/getattachment/7b0819a6-93cc4d89-8fe8-22c6ea307a

[jonm13721-bib-0005] ALBashtawy, M. , Al‐Azzam, M. , Rawashda, A. , Batiha, A. M. , Bashaireh, I. , & Sulaiman, M. (2015). Workplace violence toward emergency department staff in Jordanian hospitals: A cross‐sectional study. The Journal of Nursing Research, 23(1), 75–81. 10.1097/jnr.0000000000000075 25668738

[jonm13721-bib-0006] Aljohani, B. , Burkholder, J. , Tran, Q. K. , Chen, C. , Beisenova, K. , & Pourmand, A. (2021). Workplace violence in the emergency department: A systematic review and meta‐analysis. Public Health, 196, 186–197. 10.1016/j.puhe.2021.02.009 34246105

[jonm13721-bib-0007] Al‐Qadi, M. M. (2020). Nurses' perspectives of violence in emergency departments: A metasynthesis. International Emergency Nursing, 52, 100905. 10.1016/j.ienj.2020.100905 32818745

[jonm13721-bib-0008] American Nurses Association . (2015). Incivility, Bullying, and Workplace Violence. Retrieved from http://www.nursingworld.org/Workplace-Violence-and-Incivility-Panel

[jonm13721-bib-0009] Angland, S. , Dowling, M. , & Casey, D. (2014). Nurses' perceptions of the factors which cause violence and aggression in the emergency department: A qualitative study. International Emergency Nursing, 22(3), 134–139. 10.1016/j.ienj.2013.09.005 24168911

[jonm13721-bib-0010] Anonymous . (2017). ED violence sparks community campaign. The Lamp, 74(6), 10–11.

[jonm13721-bib-0011] Ashton, R. A. , Morris, L. , & Smith, I. (2018). A qualitative meta‐synthesis of emergency department staff experiences of violence and aggression. International Emergency Nursing, 39, 13–19. 10.1016/j.ienj.2017.12.004 29326038

[jonm13721-bib-0012] Ayranci, U. (2005). Violence toward health care workers in emergency departments in west Turkey. The Journal of Emergency Medicine, 28(3), 361–365. 10.1016/j.jemermed.2004.11.018 15769589

[jonm13721-bib-0013] Azami, M. , Moslemirad, M. , YektaKooshali, M. H. , Rahmati, S. , Soleymani, A. , Bigdeli Shamloo, M. B. , Esmaeilpour‐Bandboni, M. , Khataee, M. , Khorshidi, A. , & Otaghi, M. (2018). Workplace violence against Iranian nurses: A systematic review and meta‐analysis. Violence and Victims, 33(6), 1148–1175. 10.1891/0886-6708.33.6.1148 30573555

[jonm13721-bib-0014] Berlanda, S. , Pedrazza, M. , Fraizzoli, M. , & de Cordova, F. (2019). Addressing risks of violence against healthcare staff in emergency departments: The effects of job satisfaction and attachment style. BioMed Research International, 2019, 5430870. 10.1155/2019/5430870 31275976 PMC6558649

[jonm13721-bib-0015] Boyle, D. J. , & Hassett‐Walker, C. (2008). Individual‐level and socio‐structural characteristics of violence: An emergency department study. Journal of Interpersonal Violence, 23(8), 1011–1026. 10.1177/0886260507313966 18296572

[jonm13721-bib-0016] Buterakos, R. , Keiser, M. M. , Littler, S. , & Turkelson, C. (2020). Report and prevent: A quality improvement project to protect nurses from violence in the emergency department. Journal of Emergency Nursing, 46(3), 338, e337–344. 10.1016/j.jen.2020.02.010 32389206

[jonm13721-bib-0018] Canadian Nurses Association . (1996). Policy statement on interpersonal violence. Pearson/Prentice Hall.

[jonm13721-bib-0019] Cannavo, M. , La Torre, F. , Sestili, C. , La Torre, G. , & Fioravanti, M. (2019). Work related violence as a predictor of stress and correlated disorders in emergency department healthcare professionals. La Clinica Terapeutica, 170(2), e110–e123. 10.7417/CT.2019.2120 30993307

[jonm13721-bib-0020] Carver, M. , & Beard, H. (2021). Managing violence and aggression in the emergency department. Emergency Nurse, 29(6), 32–39. 10.7748/en.2021.e2094 34410049

[jonm13721-bib-0021] Chappell, S. (2015). The American Organization of Nurse Executives and Emergency Nurses Association guiding principles on mitigating violence in the workplace. The Journal of Nursing Administration, 45(7–8), 358–360. 10.1097/NNA.0000000000000214 26204376

[jonm13721-bib-0022] Christensen, S. S. , & Wilson, B. L. (2022). Why nurses do not report patient aggression: A review and appraisal of the literature. Journal of Nursing Management. 10.1111/jonm.13618 35403779

[jonm13721-bib-0023] Cikriklar, H. I. , Yurumez, Y. , Gungor, B. , Askin, R. , Yucel, M. , & Baydemir, C. (2016). Violence against emergency department employees and the attitude of employees towards violence. Hong Kong Medical Journal, 22(5), 464–471. 10.12809/hkmj154714 27562985

[jonm13721-bib-0024] Cohen, L. E. , & Felson, M. (1979). Social change and crime rate trends: A routine activity approach. American Sociological Review, 44(4), 588–608. 10.2307/2094589

[jonm13721-bib-0025] Copeland, D. , & Henry, M. (2017). Workplace violence and perceptions of safety among emergency department staff members: Experiences, expectations, tolerance, reporting, and recommendations. Journal of Trauma Nursing, 24(2), 65–77. 10.1097/JTN.0000000000000269 28272178

[jonm13721-bib-0026] Davey, K. , Ravishankar, V. , Mehta, N. , Ahluwalia, T. , Blanchard, J. , Smith, J. , & Douglass, K. (2020). A qualitative study of workplace violence among healthcare providers in emergency departments in India. International Journal of Emergency Medicine, 13(1), 33. 10.1186/s12245-020-00290-0 32552677 PMC7301447

[jonm13721-bib-0027] DeWall, C. N. , Anderson, C. A. , & Bushman, B. J. (2011). The general aggression model: Theoretical extensions to violence. Psychology of Violence, 1(3), 245–258. 10.1037/a0023842

[jonm13721-bib-0028] Emergency Nurses Association . (2006). Emergency Nurses Association position statement: Violence in the emergency care setting. Retrieved from https://www.ena.org/quality-and-safety/workplace-violence

[jonm13721-bib-0029] Erickson, L. , & Williams‐Evans, S. A. (2000). Attitudes of emergency nurses regarding patient assaults. Journal of Emergency Nursing, 26(3), 210–215. 10.1067/men.2000.106979 10839847

[jonm13721-bib-0030] Ferns, T. (2005). Violence in the accident and emergency department—An international perspective. Accident and Emergency Nursing, 13(3), 180–185. 10.1016/j.aaen.2005.03.005 15927470

[jonm13721-bib-0031] Ferri, P. , Silvestri, M. , Artoni, C. , & Di Lorenzo, R. (2016). Workplace violence in different settings and among various health professionals in an Italian general hospital: A cross‐sectional study. Psychology Research and Behavior Management, 9, 263–275. 10.2147/PRBM.S114870 27729818 PMC5042196

[jonm13721-bib-0032] Ferri, P. , Stifani, S. , Accoto, A. , Bonetti, L. , Rubbi, I. , & Di Lorenzo, R. (2020). Violence against nurses in the triage area: A mixed‐methods study. Journal of Emergency Nursing, 46(3), 384–397. 10.1016/j.jen.2020.02.013 32389213

[jonm13721-bib-0033] Gates, D. M. , Ross, C. S. , & McQueen, L. (2006). Violence against emergency department workers. The Journal of Emergency Medicine, 31(3), 331–337. 10.1016/j.jemermed.2005.12.028 16982376

[jonm13721-bib-0034] Gerdtz, M. F. , Daniel, C. , Dearie, V. , Prematunga, R. , Bamert, M. , & Duxbury, J. (2013). The outcome of a rapid training program on nurses' attitudes regarding the prevention of aggression in emergency departments: A multi‐site evaluation. International Journal of Nursing Studies, 50(11), 1434–1445. 10.1016/j.ijnurstu.2013.01.007 23433724

[jonm13721-bib-0035] Ghosh, M. , Twigg, D. , Kutzer, Y. , Towell‐Barnard, A. , De Jong, G. , & Dodds, M. (2019). The validity and utility of violence risk assessment tools to predict patient violence in acute care settings: An integrative literature review. International Journal of Mental Health Nursing, 28(6), 1248–1267. 10.1111/inm.12645 31454144

[jonm13721-bib-0036] Gill, M. , Fisher, B. , & Bowie, V. (2002). Violence at work causes patterns and prevention. Book Reviews. Work, Employment & Society, 17(2), 403–405.

[jonm13721-bib-0037] Gillespie, G. L. , Pekar, B. , Byczkowski, T. L. , & Fisher, B. S. (2017). Worker, workplace, and community/environmental risk factors for workplace violence in emergency departments. Archives of Environmental & Occupational Health, 72(2), 79–86. 10.1080/19338244.2016.1160861 26980080

[jonm13721-bib-0038] Gormley, M. A. , Crowe, R. P. , Bentley, M. A. , & Levine, R. (2016). A national description of violence toward emergency medical services personnel. Prehospital Emergency Care, 20(4), 439–447. 10.3109/10903127.2015.1128029 26836247

[jonm13721-bib-0039] Hamdan, M. , & Abu Hamra, A. (2015). Workplace violence towards workers in the emergency departments of Palestinian hospitals: A cross‐sectional study. Human Resources for Health, 13, 28. 10.1186/s12960-015-0018-2 25948058 PMC4435901

[jonm13721-bib-0040] Han, C. Y. , Lin, C. C. , Barnard, A. , Hsiao, Y. C. , Goopy, S. , & Chen, L. C. (2017). Workplace violence against emergency nurses in Taiwan: A phenomenographic study. Nursing Outlook, 65(4), 428–435. 10.1016/j.outlook.2017.04.003 28487095

[jonm13721-bib-0041] Harthi, M. , Olayan, M. , Abugad, H. , & Abdel Wahab, M. (2020). Workplace violence among health‐care workers in emergency departments of public hospitals in Dammam, Saudi Arabia. Eastern Mediterranean Health Journal, 26(12), 1473–1481. 10.26719/emhj.20.069 33355386

[jonm13721-bib-0042] Hassankhani, H. , Parizad, N. , Gacki‐Smith, J. , Rahmani, A. , & Mohammadi, E. (2018). The consequences of violence against nurses working in the emergency department: A qualitative study. International Emergency Nursing, 39, 20–25. 10.1016/j.ienj.2017.07.007 28882749

[jonm13721-bib-0043] Hassankhani, H. , & Soheili, A. (2017). Zero‐tolerance policy: The last way to curb workplace violence against nurses in Iranian healthcare system. Journal of Caring Sciences, 6(1), 1–3. 10.15171/jcs.2017.001 28299292 PMC5348658

[jonm13721-bib-0044] Heitmeyer, W. , & Hagan, J. (2003). International handbook of violence research (chapter II‐2.). Kluwer Academic Publishers. 10.1007/978-0-306-48039-3

[jonm13721-bib-0045] Huang, L. , Chang, H. , Peng, X. , Zhang, F. , Mo, B. , & Liu, Y. (2022). Formally reporting incidents of workplace violence among nurses: A scoping review. Journal of Nursing Management. 10.1111/jonm.13567 35213934

[jonm13721-bib-0046] Hyland, S. , Watts, J. , & Fry, M. (2016). Rates of workplace aggression in the emergency department and nurses' perceptions of this challenging behaviour: A multimethod study. Australasian Emergency Nursing Journal, 19(3), 143–148. 10.1016/j.aenj.2016.05.002 27259588

[jonm13721-bib-0047] Janice Penrod, J. E. H. (2005). Enhancing methodological clarity‐ principle‐based concept analysis. Journal of Advanced Nursing, 50(4), 403–409. 10.1111/j.1365-2648.2005.03405.x 15842447

[jonm13721-bib-0048] Jimenez, R. E. , Bachelet, V. C. , Gomolan, P. , Lefio, L. A. , & Goyenechea, M. (2019). Violence and burnout in health care emergency workers in Santiago, Chile: A survey‐based cross‐sectional study. International Emergency Nursing, 47, 100792. 10.1016/j.ienj.2019.100792 31679969

[jonm13721-bib-0049] Johnsen, G. E. , Morken, T. , Baste, V. , Rypdal, K. , Palmstierna, T. , & Johansen, I. H. (2020). Characteristics of aggressive incidents in emergency primary health care described by the Staff Observation Aggression Scale–Revised Emergency (SOAS‐RE). BMC Health Services Research, 20(1), 33. 10.1186/s12913-019-4856-9 31931790 PMC6956482

[jonm13721-bib-0050] Kennedy, M. , & Julie, H. (2013). Nurses experiences and understanding of workplace violence in a trauma and emergency department in South Africa. Health SA Gesondheid, 18(1), 663. 10.4102/hsag.v18i1.663

[jonm13721-bib-0051] Khan, M. N. , Haq, Z. U. , Khan, M. , Wali, S. , Baddia, F. , Rasul, S. , Khan, S. , Polkowski, M. , & Ramirez‐Mendoza, J. Y. (2021). Prevalence and determinants of violence against health care in the metropolitan city of Peshawar: A cross sectional study. BMC Public Health, 21(1), 330. 10.1186/s12889-021-10243-8 33568108 PMC7877048

[jonm13721-bib-0052] Kleissl‐Muir, S. , Raymond, A. , & Rahman, M. A. (2019). Analysis of patient related violence in a regional emergency department in Victoria, Australia. Australas Emerg Care, 22(2), 126–131. 10.1016/j.auec.2019.01.006 31042524

[jonm13721-bib-0053] Koller, L. H. (2016). It could never happen here: Promoting violence prevention education for emergency department nurses. Journal of Continuing Education in Nursing, 47(8), 356–360. 10.3928/00220124-20160715-06 27467310

[jonm13721-bib-0054] Kowalenko, T. , Cunningham, R. , Sachs, C. J. , Gore, R. , Barata, I. A. , Gates, D. , Hargarten, S. W. , Josephson, E. B. , Kamat, S. , Kerr, H. D. , & McClain, A. (2012). Workplace violence in emergency medicine: Current knowledge and future directions. The Journal of Emergency Medicine, 43(3), 523–531. 10.1016/j.jemermed.2012.02.056 22633755

[jonm13721-bib-0055] Kowalenko, T. , Gates, D. , Gillespie, G. L. , Succop, P. , & Mentzel, T. K. (2013). Prospective study of violence against ED workers. The American Journal of Emergency Medicine, 31(1), 197–205. 10.1016/j.ajem.2012.07.010 23000325

[jonm13721-bib-0056] Krug, E. G. , Mercy, J. A. , Dahlberg, L. L. , & Zwi, A. B. (2002). The world report on violence and health. The Lancet, 360(9339), 1083–1088. 10.1016/s0140-6736(02)11133-0 12384003

[jonm13721-bib-0057] Lancman, S. , Mangia, E. F. , & Muramoto, M. T. (2013). Impact of conflict and violence on workers in a hospital emergency room. Work, 45(4), 519–527. 10.3233/WOR-131638 23676330

[jonm13721-bib-0058] Landau, S. F. , & Bendalak, Y. (2008). Personnel exposure to violence in hospital emergency wards: A routine activity approach. Aggressive Behavior, 34(1), 88–103. 10.1002/ab.20214 17680612

[jonm13721-bib-0059] Lanza, M. L. (1984). Factors affecting blame placement for patient assault upon nurses. Issues in Mental Health Nursing, 6(1–2), 143–161. 10.3109/01612848409140887 6570953

[jonm13721-bib-0060] Lau, J. B. , Magarey, J. , & Wiechula, R. (2012). Violence in the emergency department: An ethnographic study (part II). International Emergency Nursing, 20(3), 126–132. 10.1016/j.ienj.2011.08.001 22726944

[jonm13721-bib-0061] Li, N. , Zhang, L. , Xiao, G. , Chen, J. , & Lu, Q. (2019). The relationship between workplace violence, job satisfaction and turnover intention in emergency nurses. International Emergency Nursing, 45, 50–55. 10.1016/j.ienj.2019.02.001 30797732

[jonm13721-bib-0062] Magarey, J. M. (2001). Elements of a systematic review. International Journal of Nursing Practice, 7(6), 376–382. 10.1046/j.1440-172X.2001.00295.x 11785440

[jonm13721-bib-0063] Maguire, B. J. , O'Meara, P. , O'Neill, B. J. , & Brightwell, R. (2018). Violence against emergency medical services personnel: A systematic review of the literature. American Journal of Industrial Medicine, 61(2), 167–180. 10.1002/ajim.22797 29178541

[jonm13721-bib-0064] McGuire, S. S. , Mullan, A. F. , & Clements, C. M. (2021). Unheard victims: Multidisciplinary incidence and reporting of violence in an emergency department. The Western Journal of Emergency Medicine, 22(3), 702–709. 10.5811/westjem.2021.2.50046 34125050 PMC8203007

[jonm13721-bib-0065] Morphet, J. , Griffiths, D. , Plummer, V. , Innes, K. , Fairhall, R. , & Beattie, J. (2014). At the crossroads of violence and aggression in the emergency department: Perspectives of Australian emergency nurses. Australian Health Review, 38(2), 194–201. 10.1071/AH13189 24670224

[jonm13721-bib-0066] Murray, R. M. , Davis, A. L. , Shepler, L. J. , Moore‐Merrell, L. , Troup, W. J. , Allen, J. A. , & Taylor, J. A. (2020). A systematic review of workplace violence against emergency medical services responders. New Solutions, 29(4), 487–503. 10.1177/1048291119893388 31841060 PMC8594050

[jonm13721-bib-0067] Nithimathachoke, A. , & Wichiennopparat, W. (2021). High incidence of workplace violence in metropolitan emergency departments of Thailand; a cross sectional study. Arch Acad Emerg Med, 9(1), e30. 10.22037/aaem.v9i1.1140 34027425 PMC8126349

[jonm13721-bib-0068] NSW Health . (2003). Zero tolerance: Response to violence in the NSW health workplace. NSW Department of Health.

[jonm13721-bib-0069] Ogundipe, K. O. , Etonyeaku, A. C. , Adigun, I. , Ojo, E. O. , Aladesanmi, T. , Taiwo, J. O. , & Obimakinde, O. S. (2013). Violence in the emergency department: A multicentre survey of nurses' perceptions in Nigeria. Emergency Medicine Journal, 30(9), 758–762. 10.1136/emermed-2012-201541 23038694

[jonm13721-bib-0070] Paley, J. , Cheyne, H. , Dalgleish, L. , Duncan, E. A. , & Niven, C. A. (2007). Nursing's ways of knowing and dual process theories of cognition. Journal of Advanced Nursing, 60(6), 692–701. 10.1111/j.1365-2648.2007.04478.x 18039256

[jonm13721-bib-0071] Partridge, B. , & Affleck, J. (2017). Verbal abuse and physical assault in the emergency department: Rates of violence, perceptions of safety, and attitudes towards security. Australasian Emergency Nursing Journal, 20(3), 139–145. 10.1016/j.aenj.2017.05.001 28602858

[jonm13721-bib-0072] Pich, J. , Hazelton, M. , Sundin, D. , & Kable, A. (2011). Patient‐related violence at triage: A qualitative descriptive study. International Emergency Nursing, 19(1), 12–19. 10.1016/j.ienj.2009.11.007 21193163

[jonm13721-bib-0073] Pich, J. , & Kable, A. (2014). Patient‐related violence against nursing staff working in emergency departments: A systematic review. JBI Database of Systematic Reviews and Implementation Reports, 12(9), 398–453. 10.11124/jbisrir-2014-1596

[jonm13721-bib-0074] Presley, D. , & Robinson, G. (2002). Violence in the emergency department. Nursing Clinics of North America, 37(1), 161–169. 10.1016/s0029-6465(03)00095-1 11818270

[jonm13721-bib-0075] Ramacciati, N. , Ceccagnoli, A. , & Addey, B. (2015). Violence against nurses in the triage area: An Italian qualitative study. International Emergency Nursing, 23(4), 274–280. 10.1016/j.ienj.2015.02.004 25837338

[jonm13721-bib-0076] Ramacciati, N. , Ceccagnoli, A. , Addey, B. , Lumini, E. , & Rasero, L. (2018). Violence towards emergency nurses: A narrative review of theories and frameworks. International Emergency Nursing, 39, 2–12. 10.1016/j.ienj.2017.08.004 28927973

[jonm13721-bib-0077] Ramacciati, N. , Ceccagnoli, A. , Addey, B. , & Rasero, L. (2017). Magnitude of workplace violence in emergency department: Another brick in the wall. Emergency Medicine Australasia, 29(5), 599–600. 10.1111/1742-6723.12834 28758371

[jonm13721-bib-0078] Ramacciati, N. , Ceccagnoli, A. , Addey, B. , & Rasero, L. (2018). Violence towards emergency nurses. The Italian National Survey 2016: A qualitative study. International Journal of Nursing Studies, 81, 21–29. 10.1016/j.ijnurstu.2018.01.017 29427832

[jonm13721-bib-0079] Ramacciati, N. , Gili, A. , Mezzetti, A. , Ceccagnoli, A. , Addey, B. , & Rasero, L. (2019). Violence towards emergency nurses: The 2016 Italian National Survey—A cross‐sectional study. Journal of Nursing Management, 27(4), 792–805. 10.1111/jonm.12733 30430675

[jonm13721-bib-0080] Renker, P. , Scribner, S. A. , & Huff, P. (2015). Staff perspectives of violence in the emergency department: Appeals for consequences, collaboration, and consistency. Work, 51(1), 5–18. 10.3233/WOR-141893 24939124

[jonm13721-bib-0081] Richardson, S. K. , Grainger, P. C. , Ardagh, M. W. , & Morrison, R. (2018). Violence and aggression in the emergency department is under‐reported and under‐appreciated. The New Zealand Medical Journal, 131(1476), 50–58. 29879726

[jonm13721-bib-0082] Royal College of Emergency Medicine . (2019). Retrieved from https://www.rcem.ac.uk/RCEM/News/News_2019/Emergency_Department_staff_have_the_right_to_work_in_safety.aspx

[jonm13721-bib-0083] Royal College of Nursing . (2017). Briefing on Private Members Bill: Assaults on Emergency Workers (Offences) Bill 2017‐19. The Royal College of Nursing. Retrieved from https://www.rcn.org.uk/about-us/our-influencing-work/policy-briefings/BR-0617

[jonm13721-bib-0084] Sharifi, S. , Shahoei, R. , Nouri, B. , Almvik, R. , & Valiee, S. (2020). Effect of an education program, risk assessment checklist and prevention protocol on violence against emergency department nurses: A single center before and after study. International Emergency Nursing, 50, 100813. 10.1016/j.ienj.2019.100813 32061533

[jonm13721-bib-0085] Shi, L. , Zhang, D. , Zhou, C. , Yang, L. , Sun, T. , Hao, T. , Peng, X. , Gao, L. , Liu, W. , Mu, Y. , Han, Y. , & Fan, L. (2017). A cross‐sectional study on the prevalence and associated risk factors for workplace violence against Chinese nurses. BMJ Open, 7(6), e013105. 10.1136/bmjopen-2016-013105 PMC5623406 28647719

[jonm13721-bib-0086] Sonis, J. D. , Aaronson, E. L. , Lee, R. Y. , Philpotts, L. L. , & White, B. A. (2018). Emergency department patient experience: A systematic review of the literature. Journal of Patient Experience, 5(2), 101–106. 10.1177/2374373517731359 29978025 PMC6022944

[jonm13721-bib-0087] Spelten, E. , Thomas, B. , O'Meara, P. , van Vuuren, J. , & McGillion, A. (2020). Violence against emergency department nurses; can we identify the perpetrators? PLoS ONE, 15(4), e0230793. 10.1371/journal.pone.0230793 32240231 PMC7117706

[jonm13721-bib-0088] Springer, S. , & Le Billon, P. (2016). Violence and space: An introduction to the geographies of violence. Political Geography, 52, 1–3. 10.1016/j.polgeo.2016.03.003

[jonm13721-bib-0089] Stene, J. , Larson, E. , Levy, M. , & Dohlman, M. (2015). Workplace violence in the emergency department: Giving staff the tools and support to report. The Permanente Journal, 19(2), e113–e117. 10.7812/TPP/14-187 25902352 PMC4403590

[jonm13721-bib-0090] Tadros, A. , & Kiefer, C. (2017). Violence in the emergency department: A global problem. The Psychiatric Clinics of North America, 40(3), 575–584. 10.1016/j.psc.2017.05.016 28800811

[jonm13721-bib-0091] Terry Ferns, A. C. , & Rew, M. (2005). Personal safety in the accident and emergency department. British Journal of Nursing, 14(13), 725–730. 10.12968/bjon.2005.14.13.18458 16116374

[jonm13721-bib-0092] Timmins, F. , & Timmins, B. (2021). An integrative review of waiting time, queuing, and design as contributory factors to emergency department violence. Journal of Evidence‐Based Medicine, 14(2), 139–151. 10.1111/jebm.12432 34032010

[jonm13721-bib-0093] Walker, L. O. , & Avant, K. C. (2011). Strategies for theory construction in nursing (5th ed.). Prentice Hall.

[jonm13721-bib-0094] Wax, J. R. , Pinette, M. G. , & Cartin, A. (2016). Workplace violence in health care—Its not “part of the job”. Obstetrical & Gynecological Survey, 71(7), 427–434. 10.1097/0GX.0000000000000334 27436177

[jonm13721-bib-0095] WHO . (2002). Framework guidelines for addressing workplace violence in the health sector. International Labour Office.

[jonm13721-bib-0096] WHO . (2012). Workplace violence. Retrieved from http://www.who.int/violence_injury_prevention/injury/work9/en/print.html

[jonm13721-bib-0097] Wolf, L. , Perhats, C. , Delao, A. , 2019 ENA Position Statement Committee , Brim, C. B. , Gentry, J. C. , Leaver, S. L. , Papa, A. R. , Proud, M. E. , Riwitis, C. L. , Rogers, K. S. , Stone, E. L. , Uhlenbrock, J. S. , Winger, J. , Zaleski, M. E. , 2019 ENA Board of Directors Liaison , Lee Gillespie, G. , 2019 ENA Staff Liaison , & Kolbuk, M. E. (2020). Violence and its impact on the emergency nurse. Journal of Emergency Nursing, 46(3), 354–358. 10.1016/j.jen.2020.01.005 32389208

[jonm13721-bib-0098] Wu, J.‐C. , Chen, H.‐Y. , Lee Hsieh, J. , Clinciu, D. L. , & Tung, H.‐H. (2019). Enhancing health care personnel's response to ER violence using situational simulation. Clinical Simulation in Nursing, 28, 6–14. 10.1016/j.ecns.2018.12.003

[jonm13721-bib-0099] Zafar, W. , Siddiqui, E. , Ejaz, K. , Shehzad, M. U. , Khan, U. R. , Jamali, S. , & Razzak, J. A. (2013). Health care personnel and workplace violence in the emergency departments of a volatile metropolis: Results from Karachi, Pakistan. The Journal of Emergency Medicine, 45(5), 761–772. 10.1016/j.jemermed.2013.04.049 24011477 PMC4332856

[jonm13721-bib-0100] Zhang, L. , Wang, A. , Xie, X. , Zhou, Y. , Li, J. , Yang, L. , & Zhang, J. (2017). Workplace violence against nurses: A cross‐sectional study. International Journal of Nursing Studies, 72, 8–14. 10.1016/j.ijnurstu.2017.04.002 28412581

